# The lack of correlation between symptoms and signs in patients with meibomian gland dysfunction: a secondary analysis of the multicenter, randomized controlled trial

**DOI:** 10.1186/s12886-022-02576-8

**Published:** 2022-08-28

**Authors:** Luoying Xie, Shudi Chen, Jing Hong, Xiuming Jin, Wei Chen, Bei Rong, Yun Feng, Xiaodan Huang, Jinyang Li, Wenjing Song, Lin Lin, Yu Cheng, Xiaoming Yan

**Affiliations:** 1 Department of Ophthalmology, Peking University First Hospital, Beijing, 100034 China; 2 Department of Ophthalmology, Peking University Third Hospital, Beijing, 100191 China; 3 Eye Center, The Second Affiliated Hospital of Zhejiang University School of Medicine, Hangzhou, 310009 China; 4 The School of Ophthalmology and Optometry and Eye Hospital, Wenzhou Medical University, Wenzhou, 325027 China

**Keywords:** Meibomian gland dysfunction, Symptoms, Signs, Correlation, Association

## Abstract

**Background:**

This study was performed to investigate the association between symptoms and signs in patients with meibomian gland dysfunction (MGD).

**Methods:**

Data were obtained from 122 MGD patients who were recruited for intense pulsed light therapy from November 2017 to April 2018 and the severity of their symptoms and signs at baseline were observed and recorded. Spearman correlation analyses were performed to analyze the relationships between SPEED score and signs. Subjects were divided into different subgroups based on possible influencing factors, and the differences in symptoms and signs were compared between different subgroups. Then influencing factors were controlled by regression analysis to explore the relationship between symptoms and signs and the strong factors affecting symptoms and signs.

**Results:**

Analysis of baseline data showed that SPEED scores were not correlated with TBUT, CFSS, MGYSS or any index of eyelid margin abnormality (*p* > 0.05). In addition, abnormalities of lid margins, including hyperemia, thickening, rounding, hyperkeratinization, and telangiectasia around orifices, were more likely to occur in older patients, menopausal patients, and patients living in northern China. Multiple linear regression analysis indicated that there was still no correlation between symptoms and signs (*p* > 0.05) after adjusting for influencing factors. Further analysis suggested that each influencing factor has different effects on symptoms and signs, among which menopause affects the SPEED score (R = -4.112, *p* = 0.025), and age and region have significant effects on eyelid margin abnormalities.

**Conclusions:**

In conclusion, the results demonstrated a poor correlation between symptoms and signs in MGD patients. Age, hormone, and a dry environment may influence the disease, which suggests that the severity of the disease needs to be comprehensively assessed.

## Introduction

Meibomian gland dysfunction (MGD) is a chronic ocular surface disease with symptoms that severely affect the quality of life of patients. The number of patients with meibomian gland dysfunction (MGD) has been increasing under the influence of many factors such as population aging, lifestyle changes, and widespread use of video terminals. MGD is globally widespread, with prevalence ranging from 3.5% to 69.3% and with a particularly high prevalence in Asian populations [1]. The main function of the meibomian glands is to secrete meibum, which constitutes the lipid layer of the tear film to prevent tear evaporation [2]. When the terminal duct of the meibomian gland is obstructed or the quality and quantity of meibum change, the stability of the tear film is destroyed, resulting in eye irritation or inflammatory reactions and, in severe cases, causing ocular surface damage [3].

Symptoms of eye discomfort in MGD patients include dryness, grittiness, soreness, burning sensation, blurred vision, ocular fatigue, and increased secretions. MGD is the main cause of evaporative dry eye, and while symptoms of dry eye and MGD often overlap, studies have reported that approximately 50% of MGD patients were asymptomatic [4]. In addition, some patients do not have pathological manifestations such as inflammation, or any obvious clinical signs, in which case, MGD can only be diagnosed by evaluating meibomian gland secretion [5]. This phenomenon suggests that MGD is nonobvious in some patients. However, few studies have reported whether symptoms and signs are related in MGD patients who have symptoms and signs. In this study, we will use the baseline data of patients before intense pulsed light (IPL) treatment to explore the relationship between symptoms and signs in MGD patients and provide new insights for better treatment and follow-up.

## Methods

This multicenter study was conducted at four sites in China and was approved by the ethics committee. Subjects were enrolled from November 2017 to April 2018.

### Inclusion criteria and diagnostic criteria

① Male or female patients over 18 years old; ② standard patient evaluation of eye dryness (SPEED) ≥ 6 points; ③ meibomian gland yielding secretion score (MGYSS) ≤ 12 points; ④ tear break-up time (TBUT) ≤ 10 s, (if TBUT ≤ 5 s, the inclusion criteria ⑤ are not considered); and ⑤ corneal fluorescein staining score (CFSS) ≥ 1. 

### Exclusion criteria

Patients with acute eye inflammation, active ocular infection or allergies were excluded. Patients with obvious scarring or hyperkeratinization of the eyelid were excluded. Patients suffering from OSDs (e.g., Stevens-Johnson syndrome, ocular cicatricial pemphigoid,) were excluded. Patients who used eye drops prescribed for dry eye (excluding artificial tears) within 48 h prior to enrollment, received eye surgery or eyelid surgery within 6 months, or experienced nervous paralysis were excluded. Patients with migraines and a history of head and neck radiotherapy or chemotherapy were excluded. Lactating or pregnant women were excluded.

### Research methods

The general clinical data of all patients were recorded in detail, including age, sex, menopause, surgical history, autoimmune disease history, etc. The symptoms of MGD were assessed using the SPEED questionnaire. Examination of patient signs included TBUT, CFSS, lid margin, and MGYSS.

### Standard patient evaluation of eye dryness

Symptoms were evaluated using the SPEED [6]. Subjects were asked about the frequency and severity of 4 typical symptoms of dry eye, including dryness, grittiness or scratchiness, soreness or irritation, and burning or watering. The frequency and severity of each symptom were scored on a corresponding rating scale. All scores were summed, and the total score could range from 0 to 28, with higher scores indicating more severe symptoms.

### Clinical examination

The lower palpebral conjunctiva of the subject was lightly coated with fluorescein using a moist fluorescein sodium strip (Jingmingxin Co., Ltd., Tianjin, China). The subjects were then instructed to blink 3 to 4 times to evenly distribute the fluorescein, and tear film stability was measured under a slit lamp with a cobalt blue filter. The time interval from the last time the eye was opened to the first dark spot on the tear film was recorded, and the average of 3 measurements was taken.

After measuring the TBUT, the CFSS was calculated. According to previously reported criteria [7], each quadrant of the four anatomical quadrants of the cornea was evaluated using a 3-point scale: 0 = no spot staining; 1 = less than or equal to 30 spots; 2 = more than 30 spots but no fusion; and 3 = significant fusion or ulceration. The combined CFSS for the four quadrants could range from 0 to 12.

Abnormal manifestations of eyelid margins related to MGD were observed under a slit lamp, including lid margin hyperemia, thickening, rounding, hyperkeratinization and telangiectasia around orifices.

The Meibomian Gland Evaluator (MGE; Tear Science, Inc, Morrisville, NC) was used to assess the expression of meibum and meibum traits, and the score was defined as the MGYSS. Under the slit lamp, 3 positions (nasal, medial, and temporal) of the lower eyelid were observed, with 5 glands in each position. A total of 15 gland openings were observed and the secretion at each opening was assessed. Each individual gland was evaluated using a 3-point scale: 0 = no secretion; 1 = inspissated or toothpaste-like secretion; 2 = cloudy liquid secretion; and 3 = clear liquid secretion. The score for 15 glands was recorded, with a score range of 0–45 [8].

### Statistical analysis

SPSS version 26 software (IBM, Armonk, NY, USA) was used for data analysis. The score of each clinical examination used for analysis was the average of the binocular scores from the same subject. The average SPEED score, TBUT, CFSS and MGYSS were shown as the median (interquartile range (IQR)). Spearman correlation analyses were performed to analyze the relationships between SPEED score and signs. Based on the patient's general data, the patients were subgrouped according to age, sex, menopause, and region. The Mann–Whitney tests were used to compare the differences in SPEED, TBUT, CFSS and MGYSS and the chi-square tests were performed to detect differences in the distribution of patients with eyelid margin abnormalities in different subgroups. Multiple linear regression analyses were used to analyze the relationship between symptoms and signs after controlling for influencing factors, and multiple linear regression analyses or binary logistic regression analyses were selected according to continuous variables or categorical variables to find out the main factors affecting the results. A *p*-value < 0.05 was considered statistically significant.

## Results

A total of 122 patients, with an average age of 42.2 years, were enrolled in this study, including 27 males with an average age of 38.6 years and 95 females with an average age of 43.2 years. The median SPEED score was 14.00 (11.75–18.00), median TBUT was 2.77(2.20–3.99) seconds, mean CFSS was 1.25 (0.00–2.00), and median MGYSS was 4.50(2.50–7.5). The results of the subjects after the intervention were published [9]. Here we performed statistical analysis on the baseline data.

As shown in Table 1, using Spearman correlation analyses, there was no correlation between SPEED scores and TBUT (*r* = 0.017, *p* = 0.852), CFSS (*r* = *-*0.171, *p* = 0.060), or MGYSS (*r* = *-*0.144, *p* = 0.112) in MGD patients. In addition, SPEED scores in MGD patients were not correlated with lid margin hyperemia (*r* = 0.064, *p* = 0.485), lid margin thickening (*r* = 0.091, *p* = 0.318), lid margin rounding (*r* = 0.048, *p* = 0.598), hyperkeratinization of the lid margin (*r* = *-*0.051, *p* = 0.579) or telangiectasia around meibomian gland orifices (*r* = 0.009, *p* = 0.920).

**Table 1:** Correlation analysis between SPEED scores and signs

	TBUT	CFSS	MGYSS	Lid margin hyperemia	Lid margin thickening	Lid margin rounding	Hyperkera- tinization	Telangiectasia around orifices
*r*	0.017	-0.171	-0.144	0.064	0.091	0.048	-0.051	0.009
*p*	0.852	0.060	0.112	0.485	0.318	0.598	0.579	0.920

*r*: correlation coefficient

*SPEED* standard patient evaluation of eye dryness, *TBUT* tear break-up time, *CFSS* corneal fluorescein staining score, *MGYSS* meibomian gland yielding secretion score.

In order to find the influencing factors that cause the patient's symptoms and signs to be unrelated, we performed a subgroup analysis of the data. The median age of the 122 patients was 39.5 years. Taking 40 years old as the threshold, the patients were divided into 2 groups for comparison. As shown in Table 2, there was no significant difference in SPEED, TBUT, CFSS and MGYSS between the two groups (*p* > 0.05). However, more patients in the ≥ 40-year-old group had lid margin thickening, rounding, hyperkeratinization and telangiectasia than patients in the < 40-year-old group (*p* < 0.05). In male and female patients, there was no significant difference in the severity of symptoms and signs between the two group (*p* > 0.05). Of the 95 women enrolled, 71 were premenopausal and 24 were postmenopausal. Comparison of the symptoms and signs between premenopausal women and postmenopausal women was conducted, it revealed that more postmenopausal women had lid margin hyperemia, thickening, rounding, hyperkeratinization, and telangiectasia around meibomian gland orifices than premenopausal women (*p* < 0.05). According to geographical area, the 122 patients were divided into 2 groups. As shown in Table 2, although there was no difference in SPEED, TBUT, CFSS and MGYSS between the two groups (*p* > 0.05), more patients living in the north had lid margin hyperemia, thickening, rounding, hyperkeratinization, and telangiectasia around meibomian gland orifices than patients living in the south (*p* < 0.05).

**Table 2:** Comparison of symptoms and signs of MGD patients between different subgroups

	SPEED Median (IQR)	TBUT M (IQR)	CFSS M (IQR)	MGYSS M (IQR)	a n (%)	b n (%)	c n (%)	d n (%)	e n (%)
Total (*n* = 122)	14.00(11.75–18.00)	2.77(2.20–3.99)	1.25(0.00–2.00)	4.50(2.50–7.5)	93(76.2)	40(32.8)	40(32.8)	30(24.6)	44(36.1)
Age									
< 40 years (*n* = 61)	14.00(12.00–18.00)	2.71(2.19–4.05)	1.50(0.00–2.25)	4.50(2.25–7.50)	43(70.5)	8(13.1)	5(8.2)	4(6.6)	15(24.6)
≥ 40 years (*n* = 61)	14.00(11.00–18.00)	2.84(2.26–3.82)	1.00(0.00–2.00)	5.00(2.50–7.75)	50(82.0)	32(52.5)	35(57.4)	26(42.6)	29(47.5)
*p*	0.463	0.794	0.422	0.424	0.201	<0.0001^*^	<0.0001^*^	<0.0001^*^	0.014^*^
sex									
Male (*n* = 27)	13.00(11.00–18.00)	3.14(2.62–4.23)	1.00(0.00–2.00)	5.00(2.50–9.00)	22(81.5)	8(29.6)	11(40.7)	7(25.9)	10(37.0)
Female (*n* = 95)	14.00(12.00–18.00)	2.68(2.14–3.80)	1.50(0.50–2.00)	4.50(2.50–6.50)	71(74.7)	32(33.7)	29(30.5)	23(24.2)	34(35.8)
*p*	0.736	0.059	0.176	0.302	0.611	0.818	0.357	1.000	1.000
Menopause									
No (*n* = 71)	14.00(12.00–18.00)	2.72(2.22–3.93)	1.50(0.50–2.00)	4.50(2.00–7.00)	49(69.0)	16(22.5)	13(18.3)	11(15.5)	20(28.2)
Yes (*n* = 24)	13.50(8.25–18.75)	2.62(2.00–3.42)	1.25(0.00–2.88)	4.75(2.63–6.50)	22(91.7)	16(66.7)	16(66.7)	12(50.0)	14(58.3)
*p*	0.210	0.555	0.979	0.559	0.031^*^	< 0.0001^*^	< 0.0001^*^	0.002^*^	0.013^*^
Region									
North (*n* = 69)	15.00(12.00–19.00)	2.77(2.12–4.05)	1.00(0.00–2.00)	4.50(1.50–7.00)	65(94.2)	36(52.2)	37(53.6)	28(40.6)	32(46.4)
South (*n* = 53)	14.00(11.00–17.00)	2.78(2.35–3.67)	1.50(0.00–2.25)	4.50(3.00–7.75)	28(52.8)	4(7.5)	3(5.7)	2(3.8)	12(22.6)
*p*	0.173	0.895	0.365	0.221	< 0.0001^*^	< 0.0001^*^	< 0.0001^*^	< 0.0001^*^	0.008^*^

*SPEED* Standard patient evaluation of eye dryness, *TBUT* Tear break-up time, *CFSS* Corneal fluorescein staining score, *MGYSS* Meibomian gland yielding secretion score

a: Lid margin hyperemia, b: Lid margin thickening, c: Lid margin rounding, d: Hyperkeratinization, e: Telangiectasia around orifices

^*^ p < 0.05

In the subgroup analyses, we found that age, menopause, and region may affect the patient's lid margin appearance, but not the SPEED score. Therefore, we used multiple linear regression analysis in all subjects to control for possible effects of age, sex, and region. The results found that after controlling for these three factors, there was no correlation between symptoms and signs (*p* > 0.05). In addition, multiple linear regression analysis was also used in female subjects to adjust for possible effects of age, menopause, and region. Statistical results showed that symptoms and signs were still not correlated after controlling for these three factors (*p* > 0.05) (Table 3).

**Table 3:** Multiple linear regression analysis between SPEED score and signs in all subjects or women

	Total (*n* = 122)		Women (*n* = 95)	
	Regression coefficient	*p*	Regression coefficient	*p*
TBUT	-0.119	0.705	0.069	0.850
CFSS	-0.233	0.334	-0.072	0.778
MGYSS	-0.160	0.195	-0.074	0.609
Lid margin hyperemia	0.173	0.883	-0.119	0.931
Lid margin thickening	1.650	0.144	2.330	0.063
Lid margin rounding	0.777	0.532	1.681	0.248
Hyperkeratinization	-0.952	0.429	-0.524	0.702
Telangiectasia around orifices	0.064	0.947	0.573	0.600

*SPEED* Standard patient evaluation of eye dryness, *TBUT* Tear break-up time, *CFSS* Corneal fluorescein staining score, *MGYSS* Meibomian gland yielding secretion score

In order to further explore the reasons why symptoms and signs were not related, multiple linear regression analyses and binary logistic regression analyses were used to further analyze the effect of these influencing factors on symptoms and signs in all subjects. As shown in Table 4, age may be an influencing factor of lid margin thickening (R = 0.068, *p* < 0.0001), lid margin rounding (R = 0.099, *p* < 0.0001), hyperkeratinization of the lid margin (R = 0.068, *p* < 0.0001) and telangiectasia around meibomian gland orifices (R = 0.038, *p* = 0.013). Sex may affect TBUT (R = -0.614, p = 0.049) and lid margin rounding (R = -1.851, *p* = 0.013). In addition, region may be an influencing factor of lid margin hyperemia (R = -2.671, *p* < 0.0001), lid margin thickening (R = -2.118, *p* < 0.0001), lid margin rounding (R = -2.848, *p* < 0.0001) and hyperkeratinization of the lid margin (R = -2.378, p = 0.003). The results in female subjects are shown in Table 5. Age may affect lid margin thickening (R = 0.070, *p* = 0.023), lid margin rounding (R = 0.135, *p* = 0.001), hyperkeratinization of the lid margin (R = 0.089, *p* = 0.010). Menopause had significant effect on SPEED score (R = -4.112, p = 0.025), but not on signs. Furthermore, region may affect lid margin hyperemia (R = -3.125, *p* < 0.0001), lid margin thickening (R = -2.086, *p* = 0.003), hyperkeratinization of the lid margin (R = -2.800, *p* = 0.010).

**Table 4:** Regression analysis between SPEED score and different influencing factors in all subjects

	Age		Sex (male = 0, female = 1)		Region (north = 0, south = 1)	
	Regression coefficient	*p*	Regression coefficient	*p*	Regression coefficient	*p*
SPEED	-0.050	0.134	0.396	0.706	-1.763	0.064
TBUT	0.010	0.325	-0.614	0.049*	-0.118	0.671
CFSS	0.016	0.219	0.637	0.116	0.429	0.237
MGYSS	0.017	0.494	-0.711	0.366	1.045	0.140
Lid margin hyperemia	0.010	0.669	-0.834	0.176	-2.671	< 0.0001*
Lid margin thickening	0.068	< 0.0001*	-0.276	0.652	-2.118	< 0.0001*
Lid margin rounding	0.099	< 0.0001*	-1.851	0.013*	-2.848	< 0.0001*
Hyperkeratinization	0.068	< 0.0001*	-0.685	0.293	-2.378	0.003*
Telangiectasia around orifices	0.038	0.013*	-0.315	0.521	-0.772	0.103

*SPEED* Standard patient evaluation of eye dryness, *TBUT* Tear break-up time, *CFSS* Corneal fluorescein staining score, *MGYSS* Meibomian gland yielding secretion score

^*^ p < 0.05

**Table 5:** Regression analysis between SPEED score and different influencing factors in women

	Age		Menopause (no = 0, yes = 1)		Region (north = 0, south = 1)	
	Regression coefficient	*p*	Regression coefficient	*p*	Regression coefficient	*p*
SPEED	0.063	0.267	-4.112	0.025*	-2.080	0.067
TBUT	0.021	0.204	-0.490	0.350	-0.031	0.926
CFSS	0.006	0.809	0.475	0.522	0.563	0.226
MGYSS	-0.071	0.088	2.410	0.072	0.792	0.339
Lid margin hyperemia	0.001	0.975	-0.270	0.835	-3.125	< 0.0001*
Lid margin thickening	0.070	0.023*	-0.283	0.734	-2.086	0.003*
Lid margin rounding	0.135	0.001*	-1.359	0.159	-20.909	0.997
Hyperkeratinization	0.089	0.010*	-0.958	0.284	-2.800	0.010*
Telangiectasia around orifices	0.026	0.314	0.424	0.585	-0.549	0.292

*SPEED* Standard patient evaluation of eye dryness, *TBUT* Tear break-up time, *CFSS* Corneal fluorescein staining score, *MGYSS* Meibomian gland yielding secretion score

^*^ p < 0.05

## Discussion

Clinically, there is a group of patients whose meibomian glands are obstructed and secrete viscous or even toothpaste-like secretions under pressure, but they have no symptoms or have mild symptoms; there is a group of patients with obvious symptoms of MGD and often seek medical attention, but no abnormalities are detected during the clinical examination, and these patients have to blink frequently and forcefully to alleviate the symptoms. These patients were classified as having nonobvious obstructive MGD (NOMGD) [5]. It is unclear whether this symptom-sign dissociation occurs in MGD patients who have symptoms and signs.

Our results showed that the correlation between symptoms and signs in obvious obstructed MGD patients was poor, which suggests that MGD patients may have severe symptoms, but this does not mean that their signs are also obvious, and vice versa. Postmenopausal patients are older than non-menopausal women, and older patients are more likely to have abnormal appearance of MGD. However, in our results, menopausal patients were less symptomatic, and there is a separation of symptoms and signs. We speculate that factors such as differences in patients' own sensitivity to pain and relative corneal numbness with aging or disease progression may affect patients' symptoms. In addition, although the meibomian glands may have abnormal changes such as obstruction, the residual but active meibomian glands can maintain high levels of meibomian expression [10], which may make the patient's symptoms less obvious. From this point of view, the diagnosis of MGD and the assessment of the severity of the disease should be combined with a clinical examination of various aspects and needs a comprehensive evaluation.

At present, there are few reports on correlation analyses of symptoms and signs in MGD patients. Nishant et al. [11]analyzed the correlation between symptoms and signs of 53 MGD patients, and found that although the symptom scores (Ocular Surface Disease Index (OSDI)) and the signs scores were statistically different, but not clinically significant. MGD is the main cause of evaporative dry eye, and it has been reported that the correlations between symptoms and signs in dry eye patients is also poor. Nichols et al. [12] scored the severity of MGD according to MG gland orifice obstruction and secretions, and found that the severity of MGD in patients with dry eye was not correlated with symptom frequency. They also found that there was a poor relationship between symptoms and other clinical tests, including tear meniscus height, TBUT, CFSS, phenol red thread test, Schirmer test, and rose bengal staining. Yan et al. [13] analyzed correlations between the severity of various irritation symptoms and Schirmer test scores, TBUT, CFSS, and tear film images in 126 patients with dry eye, but they found no correlation between them. In fact, dry eye has many influencing factors, and the repeatability of examination findings is poor, which may lead to poor correlation between symptoms and signs.

Dry eye is mainly characterized by an imbalance in the homeostasis of the tear film, which is determined by the lacrimal functional unit (LFU) [14]. Failure of any part of the unit may cause an imbalance in tear film homeostasis. Lin et al. [15] detected the secretion of aqueous tears in 459 symptomatic dry eye patients by the Schirmer test, and found that 38.8% of MGD patients had a Schirmer test ≤ 5 mm, suggesting that MGD patients may suffer from insufficient aqueous secretion, which indicates that some of these patients may have mixed dry eye. Since the Schirmer test or tear meniscus height examination was not performed in our study, the possibility of mixed dry eye cannot be ruled out, which may be one of the reasons for the poor correlation between symptoms and signs. In addition, Song et al. [16] conducted the partial correlation analysis between the OSDI score and the sign score of 176 dry eye patients, and found that the symptoms of the patients were positively correlated with the abnormal appearance of the eyelid margin. Inclusion criteria of this study did not include the severity of MGD, but our inclusion criteria included a meibomian gland function score of ≤ 12, which means our recruited patients had more severe meibomian gland dysfunction. This may be the reason for the different results of our study.

Since morphological changes of the eyelid margin are objective indicators of MGD, we examined the eyelid margin [17]. An abnormal eyelid margin is the external manifestation of MGD, and its internal causes are gland orifice obstruction, duct dilation, and abnormal secretion of meibum [18, 19]. In this study, we found that MGD patients in the older group were more likely to have abnormal lid margins. Age has been identified as an important factor affecting MGD [1]. In aging human and animal tissues, researchers found that the proliferation of acinar basal cells decreased, the glands were atrophied and infiltrated by inflammatory cells, and the expression of PPAR-γ was reduced or nuclear translocated [20, 21]. The abnormal changes in the eyelid margin may be related to aging. We also found that postmenopausal women were more prone to eyelid margin abnormalities. Because postmenopausal women are relatively older, age may be a factor that causes eyelid margin abnormalities. In addition, meibomian gland function has a certain relationship with sex hormones, especially after menopause. The rapid decline in sex hormone levels can induce or exacerbate MGD [22, 23]. Finally, we also noticed that abnormalities in eyelid margins were more common in MGD patients living in northern China. It is worth noting that our research was conducted in winter, the average temperature in Beijing in winter was -3 °C-0°C, and the relative humidity was 42%-43%. However, Hangzhou had an average temperature of 5 °C to 7 °C with a relative humidity of 68%-77%, and Wenzhou had an average temperature of 9–11 °C with a relative humidity of 67%-74%. The climate of Beijing was cold and dry, but the climate in Zhejiang was relatively warm and humid. Some studies have shown that a dry environment is a risk factor for MGD. Suhalim et al. [24] established a mouse model in a desiccating environment, which resulted in abnormal meibomian gland function, including an increase in the proliferation of acinar basal cells, altered lipid production, and abnormal cell differentiation. Environmental dryness or humidity may be an important reason for the difference in eyelid margins between patients living in the north and south. Although morphological changes of the eyelid margin can reflect the condition of the meibomian gland [25], patients with MGD may not have eyelid margin abnormalities, and a comprehensive assessment is needed to evaluate the presence and severity of MGD.

Our study has some limitations. Firstly, although patients undergoing eye surgery or eyelid surgery within 6 months were included in the exclusion criteria of our study. However, studies have shown that cataract surgery may cause dry eye and the condition may persist for more than a year [26, 27]. Of our subjects, 3 had cataract surgery before 6 months, which may have affected the outcome. Additionally, our inclusion criteria in our study included the MGYSS of ≤ 12, which may have caused some selectivity bias. This may have limited our search for general symptom-sign associations. Furthermore, our small sample size may be a limitation. These limitations need to be addressed in future studies.

## Conclusions

Taken together, we found that the correlation between symptoms and signs of obvious MGD in patients is poor and that age, menopause, and a dry environment may aggravate the disease. We believe that the severity of the disease needs to be comprehensively evaluated. More evidence is needed to clarify the relationship between symptoms and signs for an accurate diagnosis of the disease.

## Data Availability

The data presented in this study are available on request from the corresponding author. The data are not publicly available because the ethics committee has not approved the public availability of these data.
